# Strengthening Academic Integrity: Quantitative Insights Into Invigilation Practices in MBBS Theory Examinations

**DOI:** 10.7759/cureus.106326

**Published:** 2026-04-02

**Authors:** Rashmi Wankhade, Charuhas Akre

**Affiliations:** 1 Pathology, Government Medical College Nagpur, Nagpur, IND; 2 Community Medicine, Government Medical College Nagpur, Nagpur, IND

**Keywords:** academic integrity, examination governance, invigilation, mbbs examinations, medical education assessment

## Abstract

Background

Effective invigilation is crucial to the dependability of high-stakes medical exams. MBBS theory exams in the context of undergraduate medical education involve a significant number of candidates and require meticulous preparation to guarantee operational efficiency, security, and fairness. Despite the significance of invigilation, there is a dearth of empirical information on the effects of invigilation on academic integrity in the Indian setting.

Materials and methods

A prospective, observational, descriptive study was carried out at the Government Medical College in Nagpur during the MBBS theory exams. All registered candidates were included in the study. Structured observation and documentation formats were employed to monitor invigilation operations in real time. Candidate registration and attendance, absenteeism, academic integrity infractions (like attempts at malpractice and unauthorised use of electronic devices), operational problems (like mistakes in documentation and disruptions in procedures), medical emergencies, and the way each exam session was conducted were all included in the data that were gathered. Descriptive statistics were used to analyse the data, and the results were displayed as percentages and frequencies.

Results

A 98.1% attendance rate was achieved by 210 of the 214 registered candidates who took the test. Four candidates (1.9%) were affected by the only incident that was documented, i.e., absenteeism. Over the course of seven examination sessions, no instances of detected malpractice, unauthorised electronic or device breaches, paperwork errors, procedural disruptions, or medical/emergency incidents were seen. There was no need for invigilator interventions, as every session started on time and proceeded smoothly.

Conclusion

This study demonstrates how high attendance, seamless examination conduct, and the preservation of academic integrity during MBBS theory exams may be guaranteed by organised invigilation, adequate manpower deployment, and standardised operating standards. Exam governance and quality control in medical education may be improved by routinely recording and analysing invigilation procedures.

## Introduction

Written exams are the primary method used in undergraduate medical education to assess fundamental knowledge and determine whether to proceed to clinical training. Maintaining integrity, impartiality, and security in the evaluation process becomes more difficult as competition and stakes increase [1]. Beyond simply supervising a test, invigilation is a comprehensive professional duty that calls for crisis management abilities, ethical judgment, interpersonal awareness, and attention [2]. Invigilators play a number of vital roles. Exam security is guaranteed, applicants' identities are confirmed, academic dishonesty is discouraged, exam logistics are managed, emergencies are handled, and an atmosphere that supports an accurate evaluation of students' skills is established. This function is especially important during MBBS exams, where the stakes are quite high for patient safety and public health in addition to the academic progress of individual students [3]. This article is based on the author's personal experience as an invigilator for recent MBBS exams. It examines the theoretical foundations of successful invigilation, analyses real-world challenges and their solutions, evaluates innovative technological approaches, and provides evidence-based recommendations to enhance exam integrity while promoting student well-being.

The work of invigilators is difficult and little documented in the context of Indian MBBS exams, which involve big groups and a variety of backgrounds. Changes in invigilation procedures have been required due to issues including candidate impersonation and technology breaches. To direct continued reform initiatives, however, standardised data on operational realities, incident rates, and employee experiences is required [4]. By presenting real-world, quantitative, and qualitative data from a recent MBBS theory test, this study closes this gap and provides a basis for the advancement of practice and policy.

The objective of this study was to prospectively assess invigilation practices, operational performance, and the frequency of academic integrity-related incidents during MBBS theory examinations in a tertiary care teaching institution.

## Materials and methods

Study design

The purpose of this prospective, observational, descriptive study was to objectively assess academic dishonesty events, invigilation procedures, and operational effectiveness during MBBS theory exams.

Study setting

The study was conducted at the Government Medical College, Nagpur, during the MBBS phase II theory examination. Under standardised institutional testing norms, exams were administered in four exam halls over the course of seven sessions.

Study duration

The study was conducted during the examination period from November 29 to December 12, 2025.

Study population

All MBBS students enrolled in the theory examinations during the study period made up the study population. A total of 210 of the 214 candidates who registered showed up for the test. Since the study's goal was to obtain thorough examination-level operational data, there were no exclusion criteria.

Invigilation structure

The same invigilation structure was used for each examination session, with 14 invigilators dispersed among the four exam rooms. To guarantee proper supervision and reduce variation in examination conduct, the invigilator-to-candidate ratio was consistently maintained during all sessions.

Data collection

Pre-structured observation and documentation formats were used to prospectively gather data in real time. Operational definitions for each recorded parameter were predefined, and the same structured observation protocol was consistently applied across all examination sessions to ensure uniform data collection. All observations were recorded prospectively by the principal investigator during each examination session to maintain consistency in data collection. Every examination session involved the careful recording of all the parameters summarised in Table 1.

**Table 1:** Data collection parameters recorded during MBBS theory examinations.

Domain	Parameters recorded
Candidate details	Number of candidates registered per session. Number of candidates who appeared per session
Attendance	Absenteeism
Invigilation logistics	Number of invigilators deployed per session
Academic integrity violations	Detected malpractice attempts. Unauthorised electronic or device use
Operational issues	Documentation errors (wrong paper, extra sheets, corrections). Procedural disruptions (delayed start, seating issues, power failure)
Medical contingencies	Medical or emergency events during the examination
Qualitative observations	Overall conduct of examination sessions. Workflow efficiency

This structured approach enabled comprehensive documentation of both quantitative and observational parameters for each examination session.

Statistical analysis

Descriptive statistical methods were used to collect and analyse the data. Frequencies and percentages were used to summarise categorical variables, including attendance status, absence, and the incidence of operational or academic integrity problems. When appropriate, ranges and averages were used to depict continuous data, such as candidate age and attendance per session. To assess the uniformity of examination processes and identify any grouping of occurrences, session-wise data were closely examined. Since the study was descriptive, required a thorough enumeration of the study population, and did not seek to compare groups or test hypotheses, no inferential statistical tests were performed. Standard spreadsheet software was used for data entry and verification. To increase transparency and clarity, the results were presented in tables and graphs.

Ethical considerations

The Institutional Ethics Committee granted ethical permission (3923EC/Pharmac/GMC/NGP). All data were recorded anonymously, and no interventions were used in the study. Students' personal information was neither gathered nor shared.

## Results

Examination session and participant overview

Table 2 summarises the examination session features and candidate demographics.

**Table 2:** Examination session characteristics and candidate demographics.

Parameter	Value
Total candidates registered	214
Total candidates appeared	210
Absentees	4
Attendance rate (%)	98.1
Number of exam days/sessions	7
Number of examination halls	4
Invigilators per session	14
Total invigilator deployments	98
Average candidates per session	210
Average candidates per hall	30
Candidate age range (years)	22-23

There were 214 MBBS students registered for the theory exams, and 210 of them showed up, yielding a 98.1% attendance rate. During the exam period, four students, or 1.9% of the total, were absent. The tests were held in four different halls over the course of seven sessions. There was a strong invigilator-to-student ratio because each session was overseen by 14 invigilators, for a total of 98 invigilator assignments. With an average of 210 candidates per session and roughly 30 candidates per hall, the examinations were conducted smoothly and under efficient supervision. The candidates' ages, which varied from 22 to 23 years, suggested that the group was somewhat homogeneous.

Academic and operational incidents

The profile of operational incidents and academic integrity throughout the MBBS theory exams is shown in Table 3. The only recorded issue, which affected 1.9% of the registered candidates, was absenteeism. Three different students were absent for the final two exam sessions, while one student was absent for the first five. Even though there were four separate instances of absenteeism, they were dispersed over different sessions and featured a variety of candidates, indicating individual factors rather than systematic or operational problems. Throughout all seven exam sessions, there were no reports of attempted malpractice, unauthorised use of electronic devices, errors in documentation, interruptions to the process, or medical emergencies.

**Table 3:** The operational incident profile and academic integrity during MBBS theory examinations.

Incident type	Number	Percentage (%)
Absenteeism	4	1.9
Detected malpractice attempts	0	0
Unauthorised electronic/device breaches	0	0
Documentation errors/corrections	0	0
Procedural disruptions	0	0
Medical/emergency events	0	0
Total incidents	4	1.9

Time management and operational performance

Each examination session started properly and without any delays. Power failures, difficulty with seating, logistical challenges, or deviations from examination procedures did not occur. Effective pre-examination planning and well-coordinated invigilation are shown by the lack of procedural disturbances. Table 4 provides a summary of operational performance by session.

**Table 4:** Session-wise operational performance summary.

Session	Candidates appeared	Incidents reported	Interventions required	Comments
Session 1	210	0	0	Smooth conduct
Session 2	210	0	0	Smooth conduct
Session 3	205	0	0	Smooth conduct
Session 4	205	0	0	Smooth conduct
Session 5	210	0	0	Smooth conduct
Session 6	205	0	0	Smooth conduct
Session 7	205	0	0	Smooth conduct

The MBBS theory exams were administered over seven sessions, and Table 4 summarises the operational performance of each session. The number of applicants present varied from 205 to 210 per session. Sessions 1, 2, and 5 had 210 candidates apiece, whereas sessions 3, 4, 6, and 7 had 205 candidates apiece, indicating the distribution of candidates according to the exam papers over the course of the days. No operational problems or incidents involving academic integrity were reported during any of the sessions, and no interventions by the invigilator were needed. Every session was conducted smoothly and followed the set procedures and timetables. The fact that there were no incidents or interventions in any of the sessions shows that the invigilation processes were successfully carried out during the examination period.

Examination invigilation workflow

**Figure 1:**
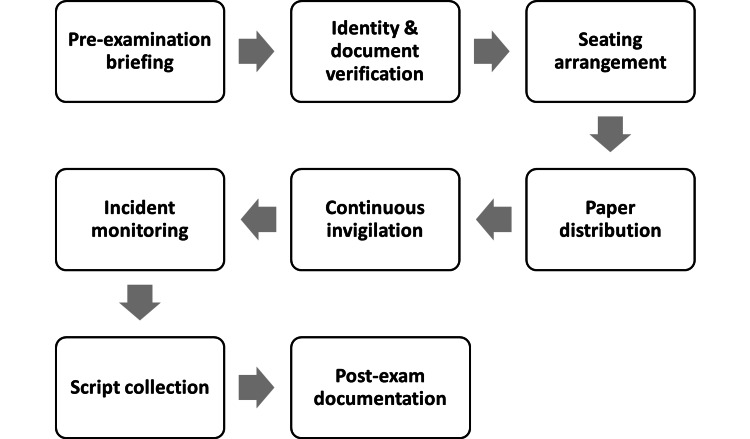
Flowchart depicting the examination invigilation process.

The structured invigilation procedure utilised for the MBBS theory exams is depicted in Figure 1. The process included pre-examination briefing, seating arrangements, coordinated paper distribution, identity and document verification, continuous invigilation, incident monitoring, organised script collection, and post-examination documentation (Figure 1).

Before the exam, a preparatory briefing was held to inform invigilators of the examination criteria, their specialised roles, hall allocations, and emergency procedures. Before being seated, candidates underwent identity and document verification at the admission points to confirm the authenticity of their admit cards and institutional credentials. After that, candidates were led to their designated seats, which had been set up ahead of time to provide proper spacing and a neat distribution throughout the testing rooms. Following the seating of all candidates, question papers were distributed in a coordinated fashion throughout all halls in compliance with the start times that had been set. As soon as the exam started, invigilators kept a close eye on candidates' behaviour and compliance with the rules. Alongside this surveillance, incident monitoring was carried out in order to quickly detect and resolve any deviations, suspected irregularities, or operational problems. Since there were no reportable occurrences during the study period, this step mostly served as a surveillance measure. Answer scripts were gathered in a systematic and sequential fashion at the end of the test to guarantee that every script was obtained and validated. Lastly, the post-examination paperwork was finished, which included creating invigilation reports, reconciling attendance, and formally delivering exam materials. This methodical, standardised procedure was used consistently during all seven exam sessions, guaranteeing consistency in the way the examinations were conducted and recorded.

Academic and operational incidents

**Figure 2:**
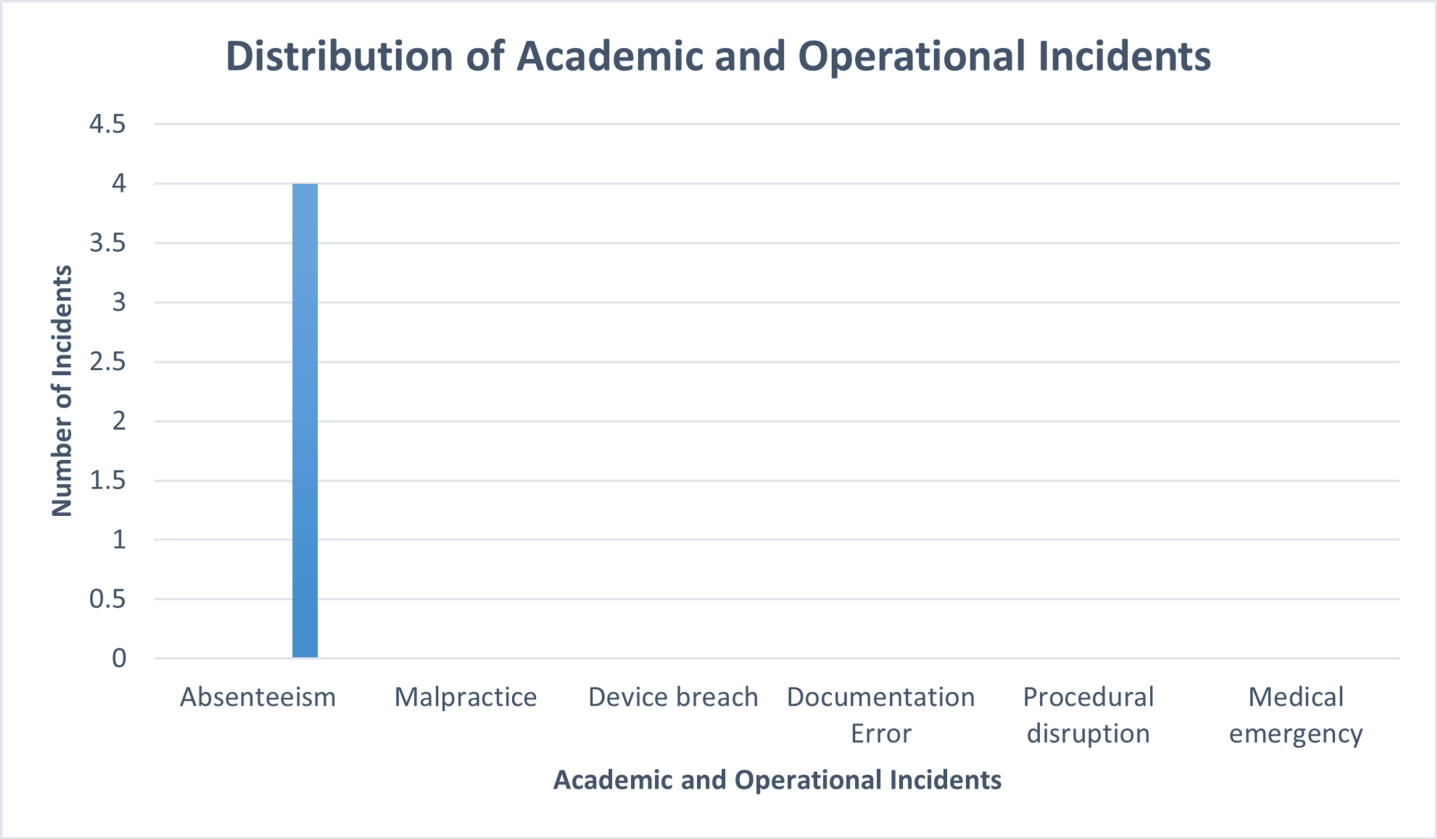
Distribution of academic and operational incidents during the examination period.

The distribution of operational and academic incidents throughout the MBBS theoretical examination period is shown in Figure 2. Out of the 214 registered candidates, four were absent, making absenteeism the only documented event. Over the course of the seven exam sessions, there were no reports of malpractice, unauthorised use of technological devices, mistakes in paperwork, disruptions in the process, or medical emergencies. As a result, every category received a zero, with the exception of absence. With absence accounting for 100% of the documented incidents (1.9% of the registered candidates), the data show a unimodal distribution of incidents. During the course of the study, no additional operational or academic integrity problems were noted.

## Discussion

The invigilation procedures and academic integrity results during MBBS theory exams at Government Medical College, Nagpur, are quantitatively analysed in this prospective observational study. Effective examination planning and execution are demonstrated by the high attendance rate of 98.1%, the lack of discovered malpractice, and the absence of operational or medical disruptions over the course of seven sessions. Absenteeism, which affected 1.9% of registered candidates, was the only documented occurrence. Compared to undergraduate medical exams in India, where absence usually varies from 2% to 5%, this absenteeism rate is favourable. In a multicentre study, Desalegn et al. observed an absence rate of 14.1%, and Joshi et al. observed an absence rate of 40.1% [5,6]. Interestingly, the study's absenteeism was dispersed across sessions and affected a variety of candidates, indicating that individual-level causes, rather than institutional or logistical problems, were at work.

Careful interpretation is necessary when there is no evidence of misconduct or unauthorised use of electronic devices. Low but persistent rates of academic misconduct in high-stakes tests are regularly reported in international literature, especially when mobile technology is involved. Technological advancements have raised the requirement for effective invigilation techniques as well as the likelihood of misconduct. These studies do, however, also show that regular enforcement, sufficient staffing, and high visibility greatly reduce misconduct [7,8]. Adequate invigilator presence and visible supervision serve as powerful deterrents to misconduct, according to Harden and Laidlaw and Frank et al. [9,10]. Structured procedures and the invigilator-to-candidate ratio upheld in this study probably aided in deterrence and compliance.

The operational efficiency found in this study is in contrast to accounts from large-scale exams that include poor recordkeeping, delayed starts, and seating mistakes. According to a study, a high rate of stress among medical students may affect their behaviour and learning [11,12]. Pre-examination briefings and well-defined invigilator roles are crucial, as seen by the lack of such disruptions in our study. On the other hand, there were no documented procedural disruptions or inaccuracies in the documentation, and every session in this study began on time. This emphasises the significance of pre-examination briefings, precise role distribution, and standard operating procedures, all of which are advised in the literature on examination governance. The prospective design and real-time data collection employed here reduce recall bias and improve internal validity when compared to research that depends on post-hoc audits or retrospective reporting. However, as invigilation-based surveillance cannot entirely rule out undiscovered intent or attempts, a shortcoming recognised in earlier academic integrity research, the lack of identified malpractice should be regarded cautiously [9,13].

This study supports national and international medical education organisations' suggestions for systematic documenting of examination procedures from a policy standpoint. Institutions can evaluate performance, find possible weaknesses, and justify budget allocation by converting normal invigilation actions into data that can be analysed [1,14]. Strong deterrents to academic misconduct have been demonstrated by adequate invigilator presence and visual surveillance, as highlighted in medical education literature [7,8]. In today's higher education, assessment design, supervisory procedures, and institutional culture all have a greater impact on academic integrity. Research shows that prospects for dishonest behaviour are greatly decreased by explicit communication of norms, thorough examination of conditions, and visible monitoring. Student trust in assessment procedures is higher, and academic misbehaviour is reported to be lower in institutions that combine preventive measures with robust invigilation systems [15]. The results of this study are compatible with these findings because there may not have been any malpractice found due to the organised invigilation process and regular presence of the invigilator.

Recommendations for examination governance

In light of the study's findings and the corpus of recent educational research, several helpful recommendations might be made. Institutions should maintain the optimal invigilator-to-candidate ratios, particularly for tests with high stakes. Pre-examination briefings ought to be required in order to ensure role clarity and protocol adherence. Even in the absence of occurrences, standard incident documentation forms should be used regularly to support auditability. Regular reviews, integration with institutional quality assurance systems, and compliance with national requirements are all highly recommended for invigilation procedures. These measures can boost exam results credibility and promote academic integrity [1,2].

Future directions and scope for further research

Future research should build on this work by using multicentric studies that encompass a range of institutional settings. CCTV analytics and digital attendance systems are examples of technology-assisted invigilation solutions that should be assessed. Future studies incorporating qualitative inputs from both students and invigilators could provide deeper insights into perceptions of examination fairness, stress, and the effectiveness of invigilation practices [2,8].

Strengths and limitations

One of the key advantages of this study is its prospective design, which reduces recall bias by having the principal investigator gather data in real time. When exam conditions remain constant over sessions, internal validity is enhanced. However, the study has a single-centre design and is limited to a single examination cycle. The distribution of genders and particular signs of psychosocial stress were not officially documented. Furthermore, the absence of detected malpractice should not be taken as a total lack of intent, which is a fundamental flaw in all invigilation-based research. Since observations were recorded by a single investigator, the possibility of observer bias cannot be completely excluded. Additionally, the possibility of a Hawthorne effect cannot be excluded, as the awareness of observation during examination sessions may have influenced the behaviour of candidates or invigilators. Future studies should focus on multicentre datasets, technology-assisted surveillance integration, and the connection between candidate outcomes and perceptions, as well as invigilation characteristics.

## Conclusions

This quantitative evaluation shows that good attendance, operational effectiveness, and the maintenance of academic integrity during MBBS theory exams depend on efficient invigilation, sufficient staffing, and standard operating procedures. The low frequency of mishaps found in this study emphasises how crucial proactive planning and methodical oversight are. Regular documentation and analysis of invigilation procedures should be encouraged as an essential part of test management in medical institutions.
